# Moving from Inpatient to Outpatient or Home Initiation of Non-Invasive Home Mechanical Ventilation

**DOI:** 10.3390/jcm12082981

**Published:** 2023-04-19

**Authors:** Mike J. Kampelmacher

**Affiliations:** 1 Department of Pulmonology, Antwerp University Hospital, Drie Eikenstraat 655, B-2650 Antwerp, Belgium; mike.kampelmacher@uza.be; 2 Laboratory of Experimental Medicine and Pediatrics, University of Antwerp, Campus Drie Eiken, Gebouw T3.30, Universiteitsplein 1, B-2610 Antwerp, Belgium

**Keywords:** home mechanical ventilation, non-invasive ventilation, outpatient initiation, home initiation, ambulatory adaptation, telemonitoring, patient selection, healthcare organization

## Abstract

Home mechanical ventilation (HMV) is an effective treatment for patients with chronic hypercapnic respiratory failure caused by restrictive or obstructive pulmonary disorders. Traditionally, HMV is initiated in the hospital, nowadays usually on a pulmonary ward. The success of HMV, and especially non-invasive home mechanical ventilation (NIV), has led to a steep and ongoing increase in the incidence and prevalence of HMV, in particular for patients with COPD or obesity hypoventilation syndrome. Consequently, the number of available hospital beds to accommodate these patients has become insufficient, and models of care that minimize the use of (acute) hospital beds need to be developed. At present, the practices for initiation of NIV vary widely, reflecting the limited research on which to base model-of-care decisions, local health system features, funding models, and historical practices. Hence, the opportunity to establish outpatient and home initiation may differ between countries, regions, and even HMV centres. In this narrative review, we will describe the evidence regarding the feasibility, effectiveness, safety, and cost savings of outpatient and home initiation of NIV. In addition, the benefits and challenges of both initiation strategies will be discussed. Finally, patient selection and execution of both approaches will be examined.

## 1. Introduction

Home mechanical ventilation (HMV) is an effective treatment for patients with chronic hypercapnic failure due to chest wall deformities, neuromuscular disease, obesity hypoventilation syndrome (OHS), and chronic obstructive pulmonary disease (COPD). As such, HMV may improve blood gases, sleep architecture, quality of life, and survival [1,2]. In the early days of HMV, back in the 1960s and 1970s, potential candidates for HMV were usually met on the intensive care unit (ICU), where they had been admitted for acute respiratory failure, often caused by a chest infection. HMV was set up in the ICU by titration of (nocturnal) ventilation guided by arterial blood gases and/or end-tidal carbon dioxide (ETCO2) measurements. Usually, it took several days before the patient was effectively ventilated and several weeks before the patient could be transferred home.

Due to the growing number of candidates for HMV as well as the increasing number of patients needing readmission to the ICU for follow-up or because of acute problems, room for HMV patients in the ICU became scarce. Following the introduction of non-invasive home mechanical ventilation (NIV) at the end of the 1980s, it became impossible to admit all these patients to the ICU, so other settings had to be sought. Initially, patients were admitted to a high-dependency or respiratory-care unit. As beds in these areas became limited, patients were admitted increasingly to the pulmonary ward. With the improvement of interfaces, ventilators, and organization of care, the number of patients initiated on NIV continued to grow further. Evidence for the effectiveness of NIV in patients with COPD and OHS caused a further increase in patients being referred for NIV [3,4]. Following the huge success of HMV, in particular NIV, even the available beds on the pulmonary wards became too scarce, and models of care that minimize the use of (acute) hospital beds had to be developed. In this narrative review, the benefits and challenges of both outpatient and home initiation of NIV will be discussed.

## 2. Outpatient Initiation of NIV

Several studies have compared outpatient initiation and inpatient initiation of NIV in clinically stable patients, predominantly with restrictive diseases (Table 1). The most frequent primary endpoints of these studies were the feasibility, safety, effectiveness, and cost savings of outpatient initiation in comparison with inpatient initiation of NIV.

### 2.1. Feasibility and Safety of Outpatient Initiation of NIV

Notwithstanding small differences with regard to location, monitoring, and guidance, all the studies confirmed that outpatient initiation of NIV was feasible and as safe as inpatient initiation [5,6,7,8,9,10,11]. Most patients needed 1–5 sessions of 2–4 h for titration of NIV and sufficient tolerance to allow ventilation throughout the entire night. Outpatient initiation was conducted by an experienced NIV clinician, usually a nurse or physician, and had a burden of work for the staff comparable to inpatient initiation [6,9]. In none of the studies did outpatients need hospitalization for adaptation to the ventilator.

### 2.2. Effectiveness of Outpatient Initiation of NIV

Ventilation was equally effective in both in- and outpatients, with equivalent improvement of nocturnal oxygen saturation, daytime arterial carbon dioxide pressure (PaCO_2_), and peak transcutaneous carbon dioxide pressure (PtcCO_2_) [5,6,7,8,9,10,11]. There was also no difference in health-related quality of life between these groups [6,7,11]. In addition, the patients’ acceptance and compliance after several months were comparable. This is essential, as survival is directly related to patient compliance [12,13]. Notably, in one study, the outpatients rated the option of being set up as an outpatient more convenient than an inpatient admission, even though they knew in advance they might need a greater number of hospital visits and would not have night-time support from the nursing staff [6]. In addition, the outpatients reported better communication and education during the initiation process than the patients initiated on NIV in the hospital or at home [10].

Furthermore, median waiting time for NIV initiation diminished more than 50% in outpatients compared with inpatients [7,8]. This is an important finding, as delaying NIV by even a few weeks may result in acute (or chronic) respiratory failure and acute admissions, particularly in patients with amyotrophic lateral sclerosis (ALS). Consequently, reduction of the waiting time for NIV to be initiated may even result in improved survival [8].

### 2.3. Cost Savings of Outpatient Initiation of NIV

Finally, in Spain, the direct costs of outpatient initiation were 44–53% lower compared with inpatient initiation, which could be attributed to a large extent to the reduced number of hospitalization days [5,7]. In the OPIP trial, the outpatient model also saved hospital days and, thereby, diminished acute bed pressure and improved access to care. However, no difference in medium-term cost-effectiveness was found [11]. In this trial, both fixed costs (NIV initiation) and variable costs (healthcare utilization) were considered. Outpatients not only required more healthcare contacts (outpatient clinic visits, phone calls, emergency home visits, and unscheduled hospital admission) but also needed more frequent adjustments of the ventilator settings following the start of NIV (62% vs. 56%). Moreover, they were prescribed an auto-titrating ventilator, which was more expensive than the NIV device used by inpatients. Finally, cost savings were reduced by the fact that, contrary to the UK, in France outpatient initiation was more expensive than inpatient initiation. In France, the inpatient initiation was performed in (relatively cheap) sleep laboratories, while in the UK, this was performed in (expensive) respiratory critical care departments [11].

## 3. Home Initiation of NIV

Around the same time that the first study comparing outpatient with inpatient initiation of NIV was started, the first trial investigating home vs. hospital initiation was begun [14]. In the last two decades, a few studies have compared home initiation to inpatient initiation of NIV in clinically stable patients, predominantly with restrictive diseases and most of them in the Netherlands (Table 2). Similar to studies on outpatient initiation, the primary endpoints of these studies were the feasibility, safety, effectiveness, and cost savings of outpatient initiation of NIV in comparison with inpatient initiation [14,15,16,17,18].

### 3.1. Feasibility and Safety of Home Initiation of NIV

All five studies confirmed that home initiation of NIV is feasible and as safe as inpatient initiation [14,15,16,17,18]. Patients started with NIV at home during the daytime under the guidance of a nurse specialized in NIV. The number of hours on NIV was gradually increased until the patients were able to use NIV during the entire night. NIV was titrated at home using pulse oximetry, transcutaneous capnography, end-tidal carbon dioxide, or blood gases. In the three studies from the Netherlands, telemonitoring equipment was used. Encrypted ventilator data, together with the data from transcutaneous capnography, were transmitted anonymously to the hospital during each night and evaluated the next day [15,16,17]. Approximately 14 days were needed to initiate NIV at home compared with 1 week in the hospital [15,16,17]. In one study, a few patients declined participation because of the expected burden for their caregivers at home [15]. However, in patients with ALS, home initiation of NIV caused a similar perceived caregiver burden as hospital initiation [18]. In none of the studies did patients initiated on NIV at home need hospitalization for adaptation to the ventilator.

### 3.2. Effectiveness of Home Initiation of NIV

The ventilation was equally effective whether initiated at home or in the hospital, with equivalent or even better improvement of nocturnal oxygen saturation, daytime PaCO_2_, and peak transcutaneous carbon dioxide pressure (PtcCO_2_) in the home initiation group [14,15,16,17,18]. Neither was there a difference in the various domains of health-related quality of life between these groups [14,15,16,17,18]. In addition, the patients’ acceptance and compliance after several months were comparable [14,15,16,17,18]. In two studies, the median waiting time for NIV initiation diminished, with 36–66% for patients initiated at home compared with inpatients [15,16], while this difference was nihil in another study [17].

### 3.3. Cost Savings of Home Initiation of NIV

The effect of home initiation of NIV on cost savings was investigated in the Netherlands only. With home initiation of NIV, the mean total of direct and indirect costs was reduced by 56–81% compared with hospital initiation [15,16,17]. This was mainly explained by a reduction of the days in the hospital.

## 4. Discussion

Although the studies discussed in this review differed in terms of country, setting, design, patient population, and number of patients, it may be concluded that, in selected patients, initiation of NIV in the outpatient or home setting appears to have important advantages for patients, HMV centres, and healthcare systems. Thus, it seems obvious to consider a change from in-hospital to outpatient or home initiation in a substantial part of referred patients. One must realize, however, that both strategies may also have drawbacks for certain patients and HMV centres. Both the advantages and disadvantages of each initiation strategy are summarized in Table 3. Given the obvious advantages of both outpatient and home initiation of NIV, the issue arises for which HMV centres is it desirable and possible to consider one or both of these initiation strategies. To answer this dilemma, five questions need to be addressed.

(1) Why initiate in the outpatient or home setting?

The most important reason for outpatient or home initiation of NIV is the shortage of beds for in-hospital initiation of NIV, both in absolute (fewer beds than before caused by shortness of staff) and relative (a higher demand for the same number of beds) terms. This is due to a steeply increased demand for NIV, in particular for patients with COPD or OHS. Consequently, the prevalence of NIV has increased considerably in the last 20 years. The estimated prevalence of HMV in 24 countries worldwide is around 7.3 (1.2–47) per 100.000 inhabitants, all disorders combined [19]. Recent data from Switzerland have shown that the prevalence has increased from 15.1 to 37.9/100.000 [2]. Likewise, the prevalence of patients with HMV in the Netherlands is estimated to have risen from 5.6 in 2001 to approximately 23.5 at present [19,20]. In addition to a reduction of the waiting time to commence NIV, other reasons for diverging from in-hospital initiation of NIV may be a reduction of the risk of acute uncontrolled ventilatory decompensation, doubts about the best setting for initiation of NIV, patient preference and convenience, and fear of infection transmission in vulnerable patients, as was recently stressed during the COVID-19 pandemic.

(2) When to consider outpatient or home initiation?

The practices for initiation of NIV vary widely, reflecting the limited research on which to base model-of-care decisions, local health system features, funding models, and historical practice. The possibility of moving to outpatient or home initiation may differ between countries, regions, and HMV centres. The local reimbursement policy may have great influence on both the desire to diverge from in-hospital initiation and the cost savings that may be achieved. For instance, in Belgium, only inpatient initiation is reimbursed, so the desire to diverge from this strategy is low from the hospital/medical point of view. 

In addition, the (social) geography of each country or region may play an important role. As the Netherlands is a small country with one of the highest population densities in the world, the travelling distance and time to hospitals is relatively small. This makes outpatient or home initiation of NIV much easier than in large countries with a low population density. However, the geography (mountains, islands) and infrastructure (highways and traffic) may play a role as well, and this may be different for each country or region. Thus, outpatient and home initiation may be easier to perform for patients living in cities than in rural or very remote areas.

Furthermore, establishing outpatient or home initiation of NIV requires enough staff and equipment. For home initiation, the staff should have extensive knowledge and experience with the many aspects of NIV to initiate this self-reliantly. In the Netherlands, most nurses working in an HMV centre are former ICU nurses, who are used to visiting patients in their home during follow-up [21]. Moreover, as inpatient initiation (with the same equipment that will be used at home) is mainly performed by these nurses, the step to outpatient or home initiation is much smaller than for nurses with less knowledge and experience, who are unfamiliar with home visits and setting up NIV. Nevertheless, moving most NIV initiations from the hospital to home will necessitate a change in the staffing model. Finally, for both outpatient and home initiation, extra equipment (ventilators, interfaces, pulse oximeters, or transcutaneous capnographs) may be needed, which requires an extra investment.

(3) Where to initiate: hospital, outpatient clinic, or home?

Elective in-hospital initiation of NIV has undoubted advantages, like the immediate availability of equipment, interfaces, and staff able to resolve any medical or technical problem that may arise. Counselling by other specialists (e.g., cardiologist, ENT specialist, gastroenterologist, or rehabilitation physician) is easy, and the input of a dietician, physiotherapist, occupational therapist, speech therapist, or psychologist may be of value. Particularly for patients with significant comorbidities; with unstable medical conditions, like acute or chronic respiratory failure; with complex care needs; needing invasive ventilation; lacking motivation; living very isolated or far away from the hospital; without transportation availability; or who are unable to perform outpatient or home initiation due to frailty or poor physical condition, anxiety or cognitive impairment, or lacking a sufficient social or professional network allowing for safe NIV initiation at home; the hospital may still be the best setting. However, inpatient initiation also has important disadvantages, like the occupation of (acute) beds, high costs, delayed initiation due to a shortage of available beds, infection risk, a rather unpleasant atmosphere, unfamiliarity with disabilities, lack of adequate resources, and, last but not least, the absence of experienced caregivers for the personal needs of these patients.

Outpatient and home initiation can avoid the inconvenience and disruption of a hospital stay. It allows patients to remain in their homes, which can be particularly important for patients who have family or job obligations. Moreover, initiating NIV at home can provide a more familiar and supportive environment for patients, as they are able to receive care from their family or caregivers in a setting where they are comfortable. Additionally, while having similar outcomes, initiation in the outpatient or home setting may be less expensive than hospital-based initiation [5,7,15,16,17].

Despite the potential benefits, there are several challenges associated with outpatient and home initiation of NIV. If the NIV equipment is not owned by the hospital, there may be logistical challenges in arranging for delivery of this equipment and ensuring that the patient and caregiver are properly trained in its use. This is very important, because it is very likely that motivation, experience, and dedication of the staff may be more essential in achieving the goals of NIV than the site at which NIV is initiated. In addition, education, training, and caregiver involvement have been shown to be important key points in facilitating the patient’s treatment experience and adherence [22]. This requires, therefore, coordination between healthcare providers, homecare providers, and the patient’s family or caregivers.

Another challenge for home initiation of NIV is the potential for complications. While NIV is generally safe, there is always a risk of complications such as pneumothorax, infection, or equipment failure. These complications may be more difficult to manage in the home setting, as patients may not have immediate access to healthcare professionals and resources.

(4) Who are suitable candidates for outpatient or home initiation?

There is the issue of patient selection. As described above, not all patients are suitable candidates for outpatient or home initiation of NIV. The patients included in studies may not always reflect real-life conditions. In the Homerun study, for example, of the 608 patients assessed for eligibility, only 96 participants were included. However, in a subsequent analysis it was speculated that in real life up to 290 (57%) of the 512 excluded patients could have started NIV at home [23]. Only patients in need of invasive ventilation, like patients with severe bulbar dysfunction, and patients with unstable medical conditions or significant comorbidities are unsuitable candidates. For patients with complex care needs who live very isolated or far away from the hospital or are unable to perform outpatient or home initiation, the hospital may still be the best setting. Nevertheless, even patients living in a nursing home, retirement home, or living congregation for disabled people could be suitable candidates for home initiation if their professional caregivers are able and willing to assist. To a lesser extent, this seems also true for patients with anxiety, cognitive impairment, and lack of motivation, but this depends very much on their social and professional support and the presence of involved caregivers who know exactly the specific needs of these patients. To become a suitable candidate, it is obvious that a thorough scan of the home environment must be performed before initiating HMV. Furthermore, healthcare providers must carefully assess each patient’s individual needs and risks to determine whether outpatient or home initiation is appropriate.

(5) How is outpatient of home initiation of HMV performed?

For outpatient initiation of NIV, a setting with one or more beds within or adjacent to the hospital is needed, preferably close to the place where all necessary equipment (particularly interfaces) is stocked. In particular, for patients with special needs, a day-care hospital setting with enough room for (family) caregivers, the opportunity of distraction (tv, radio), and enough staff to assist in daily care is convenient. For home initiation it is important that all relevant caregivers are present for the NIV initiation and training. Irrespective of the initiation site, caregivers must learn how to attach and secure the interface, how to operate the ventilator, which problems may arise, and when to call for help.

Although there is no consensus on how to monitor the initiation and titration of NIV, at minimum, gas exchange and preferably ventilator data should be followed. To monitor gas exchange, arterial blood gases are the gold standard and are quite easily performed in the outpatient setting. Though arterial blood gases may be performed in the home setting [14], they are in general unsuitable for home monitoring. Moreover, the procedure may be painful and sometimes difficult to perform, particularly in neuromuscular patients. Capillary blood gases combined with pulse oximetry may be a good alternative [24], but like arterial blood gases, in outpatients only. However, daytime blood gas measurements have been shown to be a poor reflection of the correction of nocturnal hypoventilation [25,26]. Hence, assessing the nocturnal PCO_2_ is elemental for identifying periods of nocturnal hypoventilation related to leaks, undesired respiratory events, or inappropriate settings.

PtcCO_2_ is considered reliable for continuous (nocturnal) monitoring of PaCO_2_ when patients are hemodynamically stable and devices are used by experienced staff. Several studies have reviewed the performance of commercialized capnographs and show acceptable biases and limits of agreement [25,26,27]. A review of the recent literature on PtcCO_2_ suggested that bias values up to ±1 kPa (7.5 mmHg), and limits of agreement up to ±1.33 kPa (10 mmHg), were acceptable [25]. In our centre, PtcCO_2_ is routinely performed in the home setting with relative ease and few technical problems. This may be different, however, in a home setup with telemonitoring equipment [16]. In the study by Duiverman et al., in one-third of the measurements, a technical problem impeded either reliable measurement or data transfer to the hospital [17]. The reasons for this are unclear.

## 5. Future Directions and Outlook

Although outpatient initiation of NIV may alleviate bed pressure and waiting time to NIV initiation, for most patients, home initiation of NIV seems the most advantageous strategy. This is particularly true for patients with physical disabilities who depend on adequate resources and the availability of experienced and involved caregivers for personal needs. Further studies should be undertaken to investigate the added value of this initiation strategy, particularly regarding the long-term efficiency and costs. While significant cost savings have been made in the Netherlands [15,16,17], this may be different in other countries with a dissimilar geography, population density, and healthcare system.

Telemonitoring by transmission of capnography and ventilator data may be of great value for the initiation of HMV at home, as has been shown in several trials in the Netherlands. However, before this type of monitoring can be used on a routine basis, the encountered technical problems need to be solved and better algorithms should be developed. If these improvements are achieved, then telemonitoring might assist in the initiation of NIV at home by early alerting of problems, early correction of these problems, and, thus, reaching an optimal result for the initiation of NIV at home.

If home initiation is deemed to replace inpatient initiation of NIV in the (near) future, not only is the availability of accurate, reliable, and user-friendly telemonitoring equipment needed, but also the possibility for remote adaptation of ventilator settings. While NIV is titrated as if the patient was hospitalized, home visits may be reduced to a minimum. In this way, even patients living remotely may be initiated on NIV at home. To further improve the initiation process, future studies should also investigate the additional value of ventilator data and the application of artificial intelligence for predicting problems while aiming for the best result.

## 6. Limitations of This Study

For this review, all relevant references were identified via a search carried out in Medline/PubMed until March 2023. Studies on other forms of respiratory support, like CPAP, were excluded. Based on the available literature, this review represents the personal view of the author. As such, it represents neither a meta-analysis nor a systematic review.

## 7. Conclusions

In selected patients, outpatient or home initiation of NIV appears to be safe, feasible, not inferior to in-hospital initiation, and with similar acceptance and adherence. Outpatient and home initiation of NIV may provide several benefits, including convenience for patients; reduction of the waiting time to initiate NIV, thus reducing the risk of acute uncontrolled ventilatory decompensation; and cost savings for healthcare systems. It is important, however, to carefully consider the potential challenges and risks associated with these initiation strategies, including logistical issues, technical problems, complications, and patient selection. Ultimately, the decision to initiate NIV in the outpatient or home setting should be made on a case-by-case basis, considering the patient’s individual needs and circumstances. To fine-tune this selection process and to further improve the initiation procedure, particularly in the home setting, future studies are needed.

## Figures and Tables

**Table 1:** Studies on outpatient initiation of NIV.

Study	Country	Cases No.	Design	Diseases	Sessions Needed	Cost Reduction	Primary Outcome
Luján et al., 2007 [5]	Spain	16	Prospective uncontrolled	NMD, COPD, OHS, RLD	5.5 ± 1.3	−53%	Effectiveness
Chatwin et al., 2008 [6]	UK	28	RCT	NMD, RLD	1.2 ± 0.4		Effectiveness
Pallero et al., 2014 [7]	Spain	53	RCT	NMD, OHS, RLD	2.71 ± 0.99	−44%	Effectiveness
Sheers et al., 2014 [8]	Australia	29	Prospective uncontrolled	MND	1 (0–5) days		Waiting time
Bertella et al., 2017 [9]	Italy	50	RCT	NMD	4 ± 2		Acceptance and adherence
Ribeiro et al., 2022 [10]	Portugal	235	Cross-sectional	COPD, NMD, OHS, RLD			Patient view
Murphy et al., 2023 [11]	UK and France	82	RCT	OHS	1	+6% (NS)	Costs and HRQOL

NMD = neuromuscular disease; COPD = chronic obstructive pulmonary disease; OHS = obesity hypoventilation syndrome; RLD = restrictive lung disease; RCT = randomized controlled trial; HRQOL = health-related quality of life. Data are presented as either mean ± SD or as median (range).

**Table 2:** Studies on home initiation of NIV.

Study	Country	Cases No	Design	Diseases	Primary Outcome	Cost Reduction	Monitoring
Doménech-Clar et al., 2008 [14]	Spain	42	Prospective uncontrolled	NMD, OHS, RLD	Effectiveness Daytime ΔPaCO_2_		Pulse oximetry Blood gases
Hazenberg et al., 2008 [15]	Netherlands	77	RCT	NMD, RLD	Effectiveness Daytime ΔPaCO_2_	81%	Tc Capnography Telemonitoring
Van den Biggelaar et al., 2020 [16]	Netherlands	96	RCT	NMD, OHS, RLD	Effectiveness Daytime ΔPaCO_2_	68%	Tc Capnography Telemonitoring
Duiverman et al., 2020 [17]	Netherlands	67	RCT	COPD	Effectiveness Daytime ΔPaCO_2_	56%	Tc Capnography Telemonitoring
Volpato et al., 2022 [18]	Italy	66	RCT	MND	Effectiveness Daytime ΔPaCO_2_		Pulse oximetry ETCO_2_

NMD = neuromuscular disease; OHS = obesity hypoventilation syndrome; RLD = restrictive lung disease; COPD = chronic obstructive pulmonary disease; RCT = randomized controlled trial; Tc = transcutaneous; ETCO_2_ = end-tidal carbon dioxide. Data are presented as either mean ± SD or as median (range).

**Table 3:** Advantages and disadvantages of moving from inpatient to outpatient or home initiation of non-invasive home mechanical ventilation.

Advantages	Outpatient Initiation	Home Initiation
Feasible in all disease groups: NMD, RLD, COPD, OHS	+++	+++
Fewer hospital beds needed	+++	+++
Reduced waiting time to NIV initiation	+++	+++
Potential for fewer acute admissions	+++	+++
Similar acceptance as inpatient initiation	+++	+++
Adverse effects similar to inpatient setup	+++	+++
Effectiveness equal to inpatient initiation	+++	+++
Adequate therapeutic compliance	+++	+++
Potential for cost savings	++	+++
Reduced infection risk	++	+++
Adequate resources and availability of experienced and involved caregivers for personal needs	+	+++
Familiar and supportive environment for patients	+	+++
Possibility to fulfil family or job obligations	+	+++
Patient convenience	++	+++
Patient preference	++	+++
Disadvantages	Outpatient Initiation	Home Initiation
Unsuitable for patients with unstable medical conditions, severe comorbidities, complex care needs, lack of motivation, cognitive impairment, anxiety of living far from hospital, etc.	++	+++
Implementation depends on local geography, infrastructure, and health system features	+	+++
Potential for complications, which may be difficult to manage	+	+++
Potential for higher healthcare utilization (phone calls, unscheduled hospital stays, outpatient clinic visits, and emergency home visits)	++	+++
Staff needs extensive knowledge and experience with initiation of NIV	++	+++
Need for change in staffing model	+	+++
Technical problems with (tele-)monitoring	+	+++
Investment in equipment needed	+	+++
Logistical challenges in arranging for delivery of equipment and training	+	+++
Potential for increased caregiver burden	+	+++

NMD = neuromuscular disease; RLD = restrictive lung disease; COPD = chronic obstructive pulmonary disease; OHS = obesity hypoventilation syndrome. Applicability of each item is presented as follows: + = hardly applicable, ++ = somewhat applicable and +++ = very applicable.

## Data Availability

The data presented in this study are openly available in PubMed.
